# Functional Recovery of Patient Whit Ischemic Stroke: Case Report

**Published:** 2017-11

**Authors:** Maria BECHEVA, Danail GEORGIEV

**Affiliations:** 1 Medical College, Medical University of Plovdiv, Plovdiv, Bulgaria; 2 Dept. of Biotechnology and Microbiology, Plovdiv University, Plovdiv, Bulgaria

**Keywords:** Kinesitherapy, Hemiparesis, Ischemic stroke

## Abstract

Vascular lesions of the central nervous system are one of the most important and topical problems in modern neuro-pathology. Bulgaria is among the first in morbidity and mortality from cerebrovascular diseases. Because of the significant medical and social importance of this pathology resulting in severe disabling of most survivors, we aimed to explore the effectiveness of kinesitherapeutic methodology applied in the recovery of a patient with ischemic stroke. We present a case of a 69-old patient admitted in the Neurological Ward of “St. George Hospital in Plovdiv in 2015, Bulgaria with right hemiparesis due to ischemic stroke in the basin of the left middle cerebral artery. The applied Kinesi-therapeutic methodology included various techniques like Kabat’s Bobath’s methods as well as training in equilibrium and walking. The patient obtained satisfactory results in terms of movement of the body and preserving the equilibrium, improvement of voluntary movement of the upper and lower right limb achieved via the help of some compensatory mechanisms. Despite the applied Kinesitherapy, the patient failed to gain independence in daily activities. We ascribe this satisfactory recovery only to the short period of application of kinesitherapeutic methodology. For a better recovery of patients with cerebrovascular disease, a continuous multidisciplinary approach is needed.

## Introduction

Vascular lesions of the central nervous system are one of the most important and topical problems of modern neuropathology. The alternatives to traditional hospitalization seem efficient, but they remain of limited practice because of the socio-familial constraints and the access to the health-care system (1).

Bulgaria is among the first in morbidity and mortality from cerebrovascular disease. It is the third leading cause of death in the 40 industrialized countries in the world, third only to the ischemic heart disease and the neoplastic diseases. A standardized indicator of the frequency of deaths from cerebrovascular disease shows that Bulgaria ranks fourth in Europe, with 164 deaths per 100000 population (2).

Because of the significant medical and social importance of this pathology resulting in severe disabling of most survivors, we deem it necessary to explore the effectiveness of kinesitherapeutic methodology applied in the recovery of a patient with ischemic stroke.

## Case report

We present a case of a 69**-**old patient admitted in the Neurological Ward of “St. George Hospital in Plovdiv in 2015, Bulgaria with right hemiparesis due to ischemic stroke in the basin of the left middle cerebral artery.

This was a patient with sensory aphasia and paralysis facials in the right side. The patient reported no comorbidities. He reported only the feeling of coldness in the whole right side and the “loss of power” on the same side. The patient was taking medication such as lovenox, imovan, zoloft, lexomil. The results of the kinesitherapeutic programme are published after receiving an informed consent and an approval from the ethical committee at the Medical University-Plovdiv, Bulgaria.

## Results

Kinesitherapy began on the sixth day of patient’s admission. The procedure was performed once daily. The complex lasted for 40–50 min. The average number of procedures in the clinical treatment was 10–12.

The kinesitherapeutic means included: medical massage, passive exercises of the extremities, treatment by limb exercises in position, exercises to reduce muscle tone using Bobath therapy (3), exercises to suppress pathological synergies, breathing exercises, exercises to stimulate reductions of parenthetic abdominal muscle, exercises to restore active limb movements, exercises for sitting using Bobath therapy (3), exercises for balance of seat, exercises for balance and coordination, training exercises in walking.

We conducted research on the patient’s motor functions at the beginning and end of the rehabilitation course and studied the pathological muscle tone using Brunnstrom technique (4), the strength of volitional muscle contraction on the scale of Held and Deseillignie (5), the recovery of global movements by Michel’s test (6) and the recovery of walking.

At the beginning of the study spasticity of the right upper limb and right lower limb were in second stage using Brunnstrom technique. At the end of the study is recorded on the third stage using Brunnstrom technique for right upper limb and right lower limb-strong spasticity, motor activity is entirely within the pathological synkinesis (Table 1).

**Table 1:** Beginning and results of the research of the strength of volitional muscle contraction on the scale of Held and Deseillignie in paresthetic limbs

***Muscles or muscle groups***	***Beginning***	***End***
Flexors and extensors of the fingers and wrist	0	2
Pronators in radioulnar joints	2	3
Supinators in radioulnar joints	1	2
Flexor in the elbow joint	1	3
Extensors in the elbow joint	1	2
External rotators in the shoulder joint	1	2
Internal rotators in the shoulder joint	2	3
Flexor in the shoulder joint	2	3
Extensors in the shoulder joint	2	2
Abductor in the shoulder joint	1	2
Mm. Gluteus maximus	3	4
Hip flexors	2	3
Hip abductors	1	2
Hip extensors	1	2
m. quadriceps femoris	2	3
Dorsal and plantar flexors of the foot	1	2
Flexors and extensors of toes	2	3

Initial test results o of the recovery of global movements examined by Michel’s were rating −1 on the scale/attempt for test motion – rudimentary motion/ and end results by Michel’s test were rating – 2 on scale/ makes ½ of the motion volume with effort, clumsily, motion is minimally usable/.

At the beginning of the study, the patient could not walk with aids, at the end of the study the patient was able to walk with assistance and aid only in the room. From results, we can state an improved functional status of the patient, but these results did not warrant the patient’s independence in everyday life. Throughout the course of treatment, the patients reported static pain localized in the cervical portion and tested on the scale of pain -EVA-4/10.

Although there were no signs of algoneurodystrophy in the right shoulder joint, we expected the emergence of such because the shoulder joint was painful when in motion and limited mostly to external rotation.

Violations in the surface sensitivity were difficult to study because the patient had sensory aphasia. There was established a deep sensibility disorder, investigated by the (eyes closed) position of seat, healthy upper limb forward.

## Discussion

Kinesitherapy after stroke is aimed at maximum functional recovery of the affected limbs in order to achieve independence in one’s daily life (7).

We observed an increase in muscle tone and signs of starting contractures or synkinesis, we applied passive exercises to large joints/shoulder and hip/ and gradually shifted to smaller ones. We applied passive exercises with a view to enabling the conductivity of nerve pathways, improving blood and lymphatic circulation, maintaining joint mobility and reducing the risk of formation of contractures (8).

Treatment by position was applied daily, given the spastic muscles with increased muscle tone. We observed the principles that the segments should be located opposite the pathological position. The recovery of motor functions is wholly dependent on active exercise. The deadline for commencement of active exercise is determined by the nature of the impaired brain circulation, the state of the cardiovascular system, the presence or absence of other diseases that complicate the course of the underlying disease (9).

To activate the voluntary motor activity in the initial period we used pathological reflexes for upper limb from the group of Hoffman and Trumner and for lower limb the pathological reflexes from the group of Babinski and Rosolimo. We used the Reymist phenomenon for activation of abduction in the right hip joint. We used energetic irritation on the foot of the paresthetic limb to obtain the active triple flexion.

We carried out the stimulation of the muscles of the upper limb in isolation for each unit of the limb in the horizontal plane. Stimulating the muscles was performed under the conditions of eliminating the weight of the limb not to induce excitement of spasticity (10).

After the patient acquired of the isolated active movement with assistance, we began independent performance of this movement. We avoided the use of active movements for muscle groups that are capable of increased muscle tone.

We took into account the fact that putting the patient straight early without restoring the muscle balance (between paresthetic muscles and their antagonists) could lead to a strengthening of spasticity and aggravating the muscle imbalance that hinders voluntary movements (11).

Active exercises were aimed exclusively at training in walking. Acquiring proper gait in pains largely answers the question for their independence (12). For this purpose, even in the early period before putting the patient straight we used exercises to improve the equilibrium reactions, still in supine posture, by changing the body position, then we gradually raised the patient to a sitting position. From seat position with lowered feet, we applied exercises passively upsetting the patient’s balance. This stimulated position- and-tonic reflexes (13).

Our task was not an analytical improvement in gait, but rather establishing an overall efficiency of movement of the patient to provide safe gait. We applied walking with visual control /against a mirror/, with individual phases of the servomotor cycle. The patient had to carry out the movement of the body with adapted responses.

We should not require volitional control of the patient of more than one item of gait (stride length, use of flexion in foot). Training in descending stairs was impossible because the patient was not able to carry out unilateral support on the paralyzed limb.

## Conclusion

We applied a kinesitherapeutic methodology that contributed to the satisfactory functional recovery of a patient with ischemic stroke. However, there was a deficit in terms of volitional muscle contraction, lack of selective movements, impaired locomotion and deep sensibility. This deficit was the reason for the patient’s being dependent in their daily activities on an assistant. We ascribe this satisfactory recovery only to the short period of application of the kinesitherapeutic methodology.

## Ethical considerations

Ethical issues (Including plagiarism, informed consent, misconduct, data fabrication and/or falsification, double publication and/or submission, redundancy, etc.) have been completely observed by the authors.
